# The Mechanism of Contrast-Induced Acute Kidney Injury and Its Association with Diabetes Mellitus

**DOI:** 10.1155/2020/3295176

**Published:** 2020-06-23

**Authors:** Yanfei Li, Ke Ren

**Affiliations:** Department of Radiology, Xiang'an Hospital of Xiamen University, Xiamen 361102, China

## Abstract

Contrast-induced acute kidney injury (CI-AKI) is the third most common hospital-acquired AKI after AKI induced by renal perfusion insufficiency and nephrotoxic drugs, taking great adverse effects on the prognosis and increasing hospital stay and medical cost. Diabetes nephropathy (DN) is a common chronic complication of DM (diabetes mellitus), and DN is an independent risk factor for chronic kidney disease (CKD) and CI-AKI. The incidence of CI-AKI significantly increases in patients with renal injury, especially in DM-related nephropathy. The etiology of CI-AKI is not fully clear, and research studies on how DM becomes a facilitated factor of CI-AKI are limited. This review describes the mechanism from three aspects. ① Pathophysiological changes of CI-AKI in kidney under high-glucose status (HGS). HGS can enhance the oxidative stress and increase ROS which next causes stronger vessel constriction and insufficient oxygen supply in kidney via vasoactive substances. HGS also aggravates some ion pump load and the latter increases oxygen consumption. CI-AKI and HGS are mutually causal, making the kidney function continue to decline. ② Immunological changes of DM promoting CI-AKI. Some innate immune cells and pattern recognition receptors (PRRs) in DM and/or DN may respond to some damage-associated molecular patterns (DAMPs) formed by CI-AKI. These effects overlap with some pathophysiological changes in hyperglycemia. ③ Signaling pathways related to both CI-AKI and DM. These pathways involved in CI-AKI are closely associated with apoptosis, inflammation, and ROS production, and some studies suggest that these pathways may be potential targets for alleviating CI-AKI. In conclusion, the pathogenesis of CI-AKI and the mechanism of DM as a predisposing factor for CI-AKI, especially signaling pathways, need further investigation to provide new clinical approaches to prevent and treat CI-AKI.

## 1. Introduction

Radiology and its extensive techniques play increasingly significant role in the diagnosis and treatment of many diseases, which makes the administration of iodine contrast media become very common. The terminology of acute kidney injury (AKI) caused by iodine contrast media had changed several times in these years. At first, it was called contrast-induced nephropathy (CIN). Recently, postcontrast acute kidney injury (PC-AKI) is recommended for the condition and contrast-induced kidney injury (CI-AKI) is reserved for cases in which a casual relation can be identified between contrast media administration and the kidney damage [1]. We prefer CI-AKI in this paper for much literatures use this term when published. The Contrast Media Safety Committee (CMSC) of the European Society of Urogenital Radiology (ESUR) made a most common renal function definition for CI-AKI: serum creatinine increases (sCr) ≥0.5 mg/dl (44.2 *μ*mol/L) or ≥25% from baseline within 3 days after intravascular injection of iodine contrast media, while other causes of AKI are excluded [1, 2]. After that, serum creatinine peaks at 3–5 days and drops to baseline at 10–14 days [3]. The Kidney Disease: Improving Global Outcome (KDIGO) criteria are as follows: an increase in sCr of ≥0.3 mg/dl or a sCr increase of ≥1.5–1.9 times baseline in the 48–72 h following contrast media administration [1, 4–6]. CI-AKI is the third most common hospital-acquired AKI after renal perfusion insufficiency and nephrotoxic drugs [7, 8]. It brings about adverse effect on the prognosis, increasing hospital stay and medical cost.

Various types of diabetes mellitus (DM) and their acute or chronic complications have become the common diseases threatening our lives and health, and these can be regarded as the diseases that cause secondary diseases. Although some recent reviews and studies do not emphasize DM as a direct risk factor of CI-AKI, it is still a potential predisposing factor of AKI [9, 10]. Taking a step back, diabetes nephropathy (DN) is a common chronic complication of DM, and DN is an independent risk factor for chronic kidney disease (CKD) and CI-AKI [10]. The incidence of CI-AKI significantly increases in patients with renal injury, especially in DM-related nephropathy [11]. Data showed that the incidence of CI-AKI is about 13% in nondiabetic patients and 5.7%–29.4% in diabetic patients [12].

The etiology of CI-AKI is not fully clear, and research studies on how DM becomes a predisposing factor of CI-AKI are limited. Further studies are required to answer which signaling pathways are involved in CI-AKI and their regulations on renal function. What is the potential link between high-glucose status (HGS) and CI-AKI? What are the similarities of immune response in DM and CI-AKI and so on? In this paper, the contrast media only involve iodine-based contrast media.

## 2. Pathophysiologic Changes of CI-AKI in Kidney under HGS

Current studies suggest that CI-AKI mainly occurs through two mechanisms: hypoxia damage to renal parenchyma (especially medullary hypoxia) and the toxic effects of contrast media on renal capillaries and tubules [13]. HGS may increase reactive oxygen species (ROS) [14], and then ROS bridge these two mechanisms.

Contrast media change renal hemodynamics quickly, leading to hypoxia and ischemia (or some conditions like ischemia because of temporary contrast media perfusion occurring in renal vessel). The experimental evidence related to renal hypoxia damage includes several points. ① Blood oxygen level dependent (BOLD) imaging detected an increase in deoxygenated hemoglobin in the renal medulla of animal models. ② Generation of pimonidazole adducts in the kidney was detected [15]. ③ Hypoxia-inducible factors (HIFs) accumulated [16]. Next, hypoxia and subsequent ischemia-reperfusion injury result in a large number of ROS dominated oxidation products and the formation of oxidative stress. ROS play a pivotal role in the occurrence of CI-AKI, which leads to abnormal activity of protein and enzyme, DNA structure alteration and function damage, and lipid damage [17]. The toxicity of the contrast media mainly targets at the renal vascular endothelial cells and renal tubular epithelial cells, leading to increased apoptosis and necrosis [18]. In addition, nitric oxide (NO) synthesis decreases when endothelial gets injured [19], and then arterioles and venules get more constriction than dilation, together with the weakening of antioxidation ability. Moreover, abnormal exchange of intracellular and extracellular Na^+^/Ca^2+^ and accumulation of intracellular Ca^2+^ during CI-AKI can also lead to cell damage [20]. The contrast toxicity affects activity of different cell types with a concentration-dependent change. In the meanwhile, it is found that the toxicity of ionic contrast media is higher than that of nonionic contrast media and that of hypertonic contrast media is higher than that of hypotonic contrast media [21]. The specific molecular mechanism may include the contrast media's damage to organelles through cell membrane, mitochondria impairment, efficiency of respiratory chain reduction, release of cytochrome, and induction of apoptosis via mitochondrial pathway [22]. Another round of oxidative stress ensues from ROS production after mitochondrial damage. Taken together, contrast media influence the renal microcirculation, resulting in hypoxia and ischemia-reperfusion injury and then renal tissue damage and excessive ROS production. At the same time, contrast media have toxic effect on the mitochondria of renal tubular epithelium and vascular endothelia, mediating the mitochondrial apoptosis pathway. ROS are the core part here.

DM or DN is very sensitive to the two main mechanisms above: low oxygen and toxicity [23]. In either clinical observations or animal experiments, DN is not a necessary outcome of DM but it is an important preventive target. Once DN occurs, it always means that one becomes highly prone to chronic renal failure (CRF) and end-stage renal disease (ESRD). DM makes CI-AKI happen more frequently [24] and HGS traps antioxidative system in the body accompanied by upregulation of oxidative stress [25], resulting in much more inflammation and worsened endothelial function [26]. Vice versa, it can also be considered that some pathophysiological changes in DM or DN become potential factors for the occurrence of CI-AKI, such as more oxygen consumption, the increased ROS, upregulation of endothelin and adenosine, downregulation of NO and prostaglandin (PG), and other metabolic disorders [27]. ROS generation (O_2_^−^, H_2_O_2_, and OH^−^) [28] is extraordinarily attentive because they are harmful to almost every organs and exacerbate both aging and disease processes. The production of ROS in DM and DN is mainly due to the enhanced activity of reduced nicotinamide adenine dinucleotide phosphate (NADPH) oxidase and increased mitochondrial superoxide [29]. ROS have no specific target for action. They attack lipids, proteins, and amino acids and produce unstable molecules, and the end products may bring about various metabolic effects [30]. After injection of contrast media, endothelin significantly increased in healthy subjects and those with DN and chronic kidney disease (CKD) [31]; another study found endothelin converting enzyme-1 (ECE-1) also increased [32]. Endothelin causes renal vasoconstriction, aggravating hypoxia and tubular impairment. Adenosine is an ATP metabolite that causes vasodilation in most cases but constricts vessels in kidney [33] so that it aggravates hypoxia. NO is a vasodilation substance and it gets low in renal medulla of DN [34] partly because ROS neutralize NO; as a result, vasodilation is limited. Moreover, the decrease of PG (PGE2/PGI2) synthesis makes a compound effect of renal vasoconstriction. All the vasoconstricting factors mentioned above increase, while vasodilating factors decrease, which draws down oxygen supply in kidney. It was reported that compared with normal rats, renal vasoconstriction in DM rats was more obvious [35]. Besides the effects on vasoactive substances, HGS enhances the load of certain ion pumps, increasing oxygen consumption. Some related experiments discuss this as follows. HGS increases the load of Na^+^-glucose transporter in renal tubular epithelial cells and induces more oxygen consumption [36]. Experiments were carried out in isolated medullary thick ascending limb (mTAL), which showed that the increase of ROS could enhance the synergistic transport activity of Na^+^-K^+^-2Cl^−^ [37]. Renal dysfunction can enhance Na^+^-K^+^-ATPase activity in medulla, aggravating hypoxia [38].

**Figure 1:**
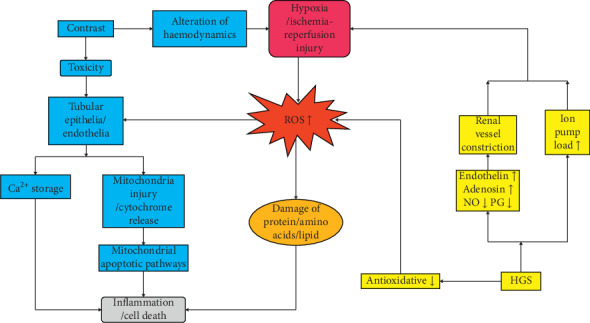
Pathophysiologic changes of CI-AKI in the kidney under HGS. Reactive oxygen species (ROS) are the core factors in both contrast-induced acute kidney injury (CI-AKI) and high-glucose status (HGS). The blue boxes comprise the main procedures of CI-AKI and the yellow ones indicate possible procedures of HGS that strengthen CI-AKI. Hypoxia and ROS production are the common alterations. The arrows link two boxes that show the pathophysiologic direction, which results in final inflammation or cell death. Some additional issues need explanation. Firstly, the vasoactive substances including but not limited to endothelin, adenosine, nitric oxide (NO), and prostaglandin (PG) because all those who are associated with vessel expanding or contracting may affect potential oxygen supply. Secondly, either damaged protein or lipid causes inflammation, apoptosis, or necrosis which are more like damage-associated molecular patterns (DAMPs) discussed later. Thirdly, the CI-AKI mainly results from tubules and endothelia injury.

In summary, HGS is a facilitating factor of CI-AKI. On the one hand, it can enhance the oxidative stress and increase ROS; on the other hand, it causes dysfunction of vasoactive substances, stronger vessel constriction in the kidney, and insufficient oxygen supply. It also aggravates some ion pump load, increasing oxygen consumption. CI-AKI can also aggravate the pathological process of DM and DN, and they are mutually causal, making the kidney function continue to decline (Figure 1).

## 3. Immunological Changes of DM Promote CI-AKI

Clinically, contrast media can trigger a series of adverse reactions, such as nausea, vomiting, urticaria, bronchospasm, and hypotension, but the mechanisms of these reactions have not been fully understood nor have been clear whether these side effects have associations with CI-AKI. The specific immune changes in HGS which tend to trigger CI-AKI is ambiguous.

### 3.1. Cytokines

Various cytokines upregulate in diabetics and are detectable in plasma [39]. Cytokines' upregulation positively correlates with the progress of DM [40] and urine protein [41]. The involved cytokines are TNF-*α* (tumor necrosis factor-alpha), IL-1 (interleukin-1), IL-18, TGF-*β* (tumor growth factor-beta), IFN-*γ* (interferon-gamma), IL-6, and IL-33. The facilitation of their effects on CI-AKI is direct or indirect.

#### 3.1.1. TNF-Alpha and IL-1

Decades ago, Hasegawa reported that, compared with normal rats, cocultured renal basement membrane of DM rats with macrophages produced much more TNF-alpha and IL-1 [42], stimulating inflammation. TNF-alpha is produced mainly by monocytes-macrophages, T cells, and also by renal cells [43]. TNF-alpha takes direct toxic effects on renal cells inducing damage, apoptosis, and necrosis [44, 45]. In addition, TNF-alpha induces ROS generation in renal cells, intensifying oxidative stress [46], and then oxidative stress disrupts antioxidant system and worsens immune function [47]. The toxicity of these two kinds of cytokines and ROS promotes CI-AKI.

#### 3.1.2. IL-18, TGF-Beta, and IFN-Gamma

Various cells can release IL-18, such as monocytes-macrophages and T cells. The tubular epithelia of patients with DN excrete more IL-18 and stimulate the release of IFN-gamma [48] and then activate mitogen-activated protein kinase (MAPK) pathway. Lee et al. found that certain extracted flavonoid from *Artemisia argyi* was able to inhibit MAPK phosphorylation and perform antiapoptosis action in CI-AKI [49]. This provided a new indirect evidence that IL-18 may promote CI-AKI. Another earlier study reported that TGF-beta1 is an upstream member of IL-18 [50] and plays a key role in kidney fibrosis and antifibrosis, which has strong association with CKD and ESRD. In the murine model with CKD, contrast media activated TGF-beta/pSMAD3 signaling pathway and upregulated connective tissue growth factor (CTGF), matrix metalloproteinase 9 (Mmp-9), and type IV collagen, thereby inducing cellular death [51]. A deduction that TGF-beta works the same way in DM/DN was made. Yaribeygi et al. made a summary about the effects of IL-18 in DN [52], considering that IL-18 has connections with different cytokines and pathways that promote DN and the underlying mechanisms including mesangial proliferation and glomerular fibrosis, intensifying oxidative stress and induction of apoptosis or necrosis. It is not hard to notice that these effects are closely related to the pathophysiological changes of CI-AKI.

#### 3.1.3. IL-6

DN is characteristic of glomerular interstitial fibrosis. In DM patients, IL-6 in patients with DN is higher than that in DM without DN [53]. IL-6 promotes mesangial proliferation, makes endothelia more penetrable, and increases fibronectin [54]. These are pathological features of renal fibrosis. As a special AKI, CI-AKI also probably develops to CKD if treatment was not enough, which means an increasing possibility of fibrosis.

#### 3.1.4. IL-33

Onk et al. had found that IL-33 levels were much higher in the kidney and serum of rats with DM, and they increased further in DM rats which were given contrast media [55]. Thereafter, they found melatonin could reverse this process and attenuate renal damage [55], which perhaps relates to the immune regulation, antiapoptosis, and antioxidative stress of melatonin. In a clinical study, Oweis et al. analyzed serum from 202 patients undergoing coronary angiography and found that serum IL-33 can be regarded as a predictor of CI-AKI [56].

#### 3.1.5. IL-22

Different from the above cytokines, IL-22 may inhibit inflammation and alleviate renal impairment in DM and CI-AKI. Wang et al. [57] reported that IL-22 decreased in DM patients and the DM model. The upregulation of IL-22 attenuated renal damage in the DM model and relieved the overexpression of fibronectin and type IV collagen and then alleviated renal fibrosis and albuminuria [57]. The suppression of NOD-like receptor pyrin 3 (NLRP3)/caspase-1/IL-1beta is likely to be involved in these series of reactions.

**Figure 2:**
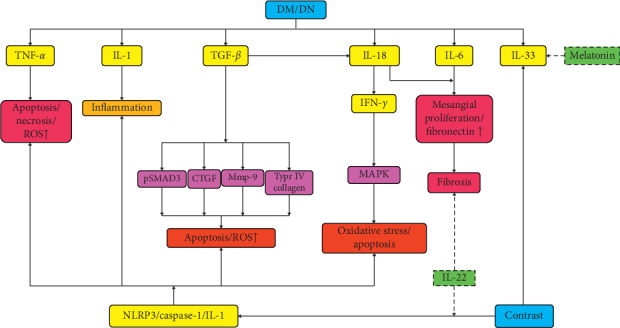
Cytokines in HGS and CI-AKI. This concentrates mainly on how cytokines are affected by diabetes mellitus (DM)/diabetes nephropathy (DN) or high-glucose status (HGS) and contrast media, which further delineates underlying mechanisms of why DM/DN is a risky factor for contrast-induced kidney injury (CI-AKI). The solid arrows mean increasing, promoting, or activating, and the dotted arrows denote decreasing, weakening, or inactivating. The yellow boxes give the associated cytokines, and most of them increased by DM/DN and then caused apoptosis or necrosis directly or pathologic alteration such as fibrosis and inflammation. TGF-beta and IL-18 cause oxidative stress or apoptosis via some special molecules involved in certain signaling pathways. NOD-like receptor pyrin 3 (NLRP3) is involved in CI-AKI, which is discussed in detail later. Cytokines that relieve renal injury are limited. IL-22 counteracts fibrosis and suppresses NLRP3/caspase-1/IL-1beta. Melatonin alleviates renal damage by reversing IL-33 increase. CTGF, connective tissue growth factor; IFN-*γ*, interferon-gamma; IL-1/6/18/22/33, interleukin-1/6/18/22/33; MAPK, mitogen-activated protein kinase; Mmp-9, matrix metalloproteinase 9; NLRP3, NOD-like receptor pyrin 3; ROS, reactive oxygen species; TGF-*β*, tumor growth factor-beta; TNF-*α*, tumor necrosis factor-alpha.

Together, multiple cytokines implicate changes in both DM and CI-AKI, and most of them are against normal renal function. IL-22 is an exception that relieves renal inflammation, and IL-33 in serum is a potential predictor for CI-AKI (Figure 2).

### 3.2. Pattern Recognition Receptors and Immune Cells

In studies of DN patients and animal models, about five kinds of cells take part in renal pathogenic effect [58]: neutrophils, lymphocytes, macrophages, dendrites, and mast cells. These cells infiltrate into kidney and release proinflammatory factors, causing degradation and phagocytosis of necrotic cell fragments, and promote fibroblast proliferation and renal fibrosis. Except lymphocytes, the other four types of cells are important members of the innate immune system. Innate immune cells express pattern recognition receptors (PRRs), such as toll-like receptors (TLRs) and NOD-like receptors (NLRs), to combine with specific ligands, such as damage-associated molecular patterns (DAMPs) or pathogen-associated molecular patterns (PAMPs), to trigger immune responses. The previous concentrations of immune cells in DM were mostly on islet cells and fatty tissue and subsequent results are injuries of islet cells (Type 1 Diabetes Mellitus, T1DM), insulin resistance (Type 2 Diabetes Mellitus, T2DM), or obesity [59]. The studies on molecular patterns and receptors in CI-AKI are limited.

At first, HGS induces TLR overexpression, such as TLR2 and TLR4 in fatty tissue [60], retina [61], endothelia of coronary arteries [62], and renal vessels [63]. A recent study showed that oxidative stress (ROS and H_2_O_2_) promoted expression of TLR2 and TLR4 in human periphery monocytes and upregulated sorts of cytokines, such as IFN-gamma, IL-1beta, IL-6, and so on, consisting of some cytokines in DM. This finding provided new evidence of why DM increases CI-AKI incidence [64]. Some other studies indicated that in non-contrast-induced AKIs, TLRs interplay with DAMPs or PAMPs, resulting in immune reactions or mediating immune responses [65]. PAMPs originate from certain pathogens and usually exist but not morbific in healthy human body, so discussions on DAMPs have more connection with CI-AKI. In DM and DN, DAMPs stem from damaged or dead cells and they could be metabolites of ATP, DNA, or harmful particles such as uric acid crystals or can be generated by radiation [66]. DAMPs get exposed to the immune system via cytolysis, cellular excretion, or enzyme matrix releasing [67]. Heat shock protein (HSP) and high mobility group box-1 protein (HMGB-1) are the most common DAMPs in AKI [68]. Moreover, on the one hand, innate immune cells express TLRs to recognize DAMPs, activating some signaling pathways, such as necrosis factor-kappa B (NF-*κ*B) and MAPK, and promoting cytokine release. Some of these factors may be involved in CI-AKI. On the other hand, NLRs participate in formation of some signaling complex and cause damage. For example, NLRP3 inflammasome is a complex which can activate caspase-1 and then promotes IL-1beta, IL-18, and IL-33 mature and releasing [69] (Figure 2). A recent research from Lau et al. showed that several steps accompanied in immune surveillance of CI-AKI, and one of them was NLRP3-dependent inflammatory response [70]. Contrary to the above results, IL-22 can alleviate renal injury and fibrosis in DN by inhibiting the NLRP3/caspase-1/IL-1beta inflammatory response pathway [57]. Yaribeygi et al. concluded that resolvins inhibited the formation of NLRP3 inflammasome and NF-*κ*B pathway and recruited inflammatory cells, thereby relieving oxidative stress and alleviating DN [71]. These consequences gave indirect evidence to the participation of NLRP3 in CI-AKI.

**Figure 3:**
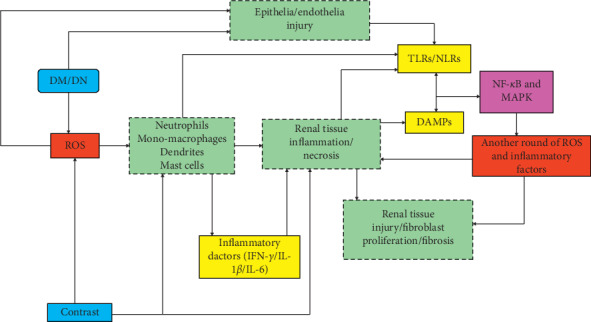
PRRs in CI-AKI and DM/DN. Various kinds of cells, especially immune cells, respond to diabetes mellitus (DM)/diabetes nephropathy (DN), and contrast media. The light-green dotted boxes implicate the possible cells in the process. Solid arrows head for the response direction. Toll-like receptors (TLRs)/NOD-like receptors (NLRs) and damage-associated molecular patterns (DAMPs) play an important role in innate immunity and answer for contrast media or high-glucose status (HGS). Immune cells affect renal tissue mainly through inflammation and cytokines. NF-kappa B and MAPK are the possible pathways between pattern recognition receptors (PRRs) and another round of hit on the kidney. Reactive oxygen species (ROS) and cytokines (inflammatory factors) are the key actors. The aftermath is renal fibrosis and injury and then cell death. IFN-*γ*, interferon-gamma; IL-1*β*/6, interleukin-1*β*/6; MAPK, mitogen-activated protein kinase; NF-*κ*B, necrosis factor-kappa B.

Now, we can see that some innate immune cells and PRRs in DM and/or DN may respond to some DAMPs formed by CI-AKI, secrete cytokines, and aggravate or inhibit inflammation. These effects overlap with some pathophysiological changes in hyperglycemia, providing new research ideas in DM that promotes CI-AKI and the related mechanisms (Figure 3).

## 4. Signaling Pathways Related to CI-AKI and DM

The pathological changes of renal cells in CI-AKI are under control of different signaling pathways. The toxicity of contrast media, hypoxia, and elevated ROS, together with immune reactions or inflammatory responses, can increase cellular apoptosis and inactivate transcription and translation of renal cells. By far, the involved signaling pathways have been found as ① Bax/Bcl2-caspase-3/9, ② PKB/mTOR/p70S6, ③ PKB/FoxO, ④ p38 MAPK, ⑤JNK, and ⑥ NF-kappa B. The protection pathways may include ①, ②, ④, and ⑥. And the above pathways related to DM/DN are ③, ④, ⑤, and ⑥.

### 4.1. Signaling Pathways Induce CI-AKI

Ioversol can induce apoptosis of LLC-PKI renal epithelia. The upregulation of Bax and downregulation of Bcl-2 with more expression of caspase-3 and caspase-9 can be detected. This process can be reversed by cAMP through PKA-dependent cAMP-responsive element binding protein (CREB) phosphorylation [72–74]. Andreucci et al. found that in HK-2 renal epithelia, diatrizoate and iomeprol induced p38 mitogen-activated protein kinase (MAPK), c-Jun N-terminal kinase (JNK), and NF-*κ*B phosphorylation, resulting in inflammation and apoptosis, and diatrizoate had a stronger effect [75]. After that, this group found iomeprol and iodixanol made protein kinase B (PKB or Akt) dephosphorylation in HK-2 and inactivated the targets at downstream. The activity of p70S6 kinase gets weaker and the protein synthesis is blocked, thereby causing cell death. Meanwhile, iomeprol causes dephosphorylation of forkhead box O3a (FoxO3a) and cell death [76].

These *in vitro* experiments demonstrate to some extent the harmful effects of contrast media on kidney cells and indicate possible signaling pathways, but further experiments validation *in vivo* is necessary and make them targeted pathways for the prevention and treatment of CI-AKI in clinic.

### 4.2. The Possible Targeting Pathways in Relieving CI-AKI

Antioxidant N-acetylcysteine (NAC) is getting more attention because of its ability to alleviate CI-AKI. N-acetylcysteine amide (NACA) is an amide of NAC, and it can mitigate oxidative stress and reduce apoptosis more effective than NAC through p38 MAPK [77]. In ischemia tissue, phosphatidylinositol-3 kinase/serine-threonine kinase B (P13K/Akt) and its downstream molecules play a role in cell protection and survival [78]. Salvianolic B can relieve oxidative stress and inflammation via PI3K/Akt/nuclear factor-E2-related factor 2 (Nrf2) and improve renal function in CI-AKI [79]. In another *in vivo* study, the author found that sulforaphane (SFN) increased the expression of heme oxygenase-1(HO-1) and then activated cells to decrease ROS, thereby relieved CI-AKI [80].

All the above pathways are involved in reducing oxidative stress and ROS production. It has been further confirmed that oxidative stress and ROS are the core factors of CI-AKI, making various pathways that can reduce oxidative stress become targets for the treatment of CI-AKI.

### 4.3. Various Pathways in DM May Intensify CI-AKI

HGS affects various signaling pathways in different cells and is complicated. For example, FoxO1 overexpress in cardiomyocyte with DM, resulting in cardiac metabolic disorders, activation of caspases, and increased mitochondrial apoptosis [81]. In addition, high glucose activates JNK and p38 MAPK and promotes the apoptosis of annulus fibrosus cell in intervertebral disc [82]. The activation of these pathways in diabetic kidneys remains to be investigated, and the results are likely to be positive due to the widespread presence of these signaling pathways.

At present, some scholars have associated ROS with a series of signaling pathway activation in renal studies related to DM and hyperglycemia, such as PKC and NF-kappa B [83]. These pathways can lead to renal fibrosis and renal function decline [84]. High glucose activates intracellular protein kinase C (PKC), which regulates vasoconstriction, cell growth, angiogenesis, cytokine activity, and leukocyte adhesion [85]. PKC can also increase the expression of TGF-*β*, leading to extracellular matrix aggregation, and promote renal interstitial fibrosis [86].

The NF-kappa B plays a critical role in regulating inflammation, as well as the expression of angiotensin, cytokines, and adhesion molecules [87, 88], and Nrf2 can suppress NF-kappa B with negative feedback [89]. Khaleel et al. demonstrated that Nrf2/HO-1 pathway protected CI-AKI in diabetic rats and further demonstrated the protective effect of sulforaphane on CI-AKI [90].

**Figure 4:**
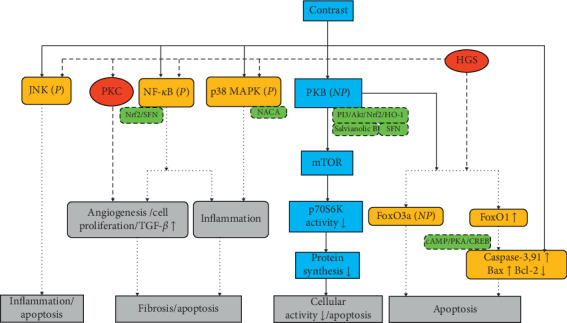
Signaling pathways associated with CI-AKI and HGS in tubular epithelia contrast media trigger multiple signaling pathways in tubular epithelia, and the responses become more dynamic in HGS. The red circles show the pathways high-glucose status (HGS) caused directly, the blue boxes only show contrast-induced pathways, and the yellow rounded boxes give the common pathways which are likely to be the mechanisms firstly exist in diabetes mellitus (DM)/diabetes nephropathy (DN) and then worsen contrast-induced kidney injury (CI-AKI). The green dotted boxes contain substances or the target that may relieve CI-AKI. All the pathways pointed to apoptosis, inflammation, and fibrosis, which is the same like the former three figures. CREB, cAMP-responsive element binding protein; FoxO, forkhead boxO; HO-1, heme oxygenase-1; JNK, c-Jun N-terminal kinase; MAPK, mitogen-activated protein kinase; mTOR, mammalian target of rapamycin; NACA, N-acetylcysteine amide; NF-*κ*B, necrosis factor-kappa B; Nrf2, nuclear factor-E2-related factor 2; P13K, phosphatidylinositol-3 kinase/serine-threonine kinase B; PKA/B/C, protein kinase A/B/C; SFN, sulforaphane; P, phosphorylation; NP, dephosphorylation.

The pathogenesis of CI-AKI based on DM is very complex, and various signaling pathways may be involved. The interaction of various pathways overlaps and promotes each other. And the transcription factors, Nrf2 and NF-*κ*B, work as redox switches responding to ROS [17]. The overall consequence is aggravation of renal inflammation and cellular apoptosis and kidney fibrosis (Figure 4).

## 5. Summary

The mechanism of how DM works on CI-AKI is not fully clear, nor does CI-AKI itself. Current opinions mainly focus on hypoxia of renal parenchyma and renal toxicity. ROS are the key factors in CI-AKI, and, HGS, especially the changes of vascular active substances intensifying renal injury, can promote the occurrence of CI-AKI. In addition, the alterations of immune response in CI-AKI process are worth discussing. Quite a few studies have found regular changes of some cytokines in CI-AKI, and the effects of these cytokines seem to be similar to those on renal function in DM. It is worth noting that we can hardly make clear differentiation on HGS, DM, or DN when discussing specific points because it is hard to define a completely same condition with various laboratory controls. Nevertheless, it is essential to differentiate them when back to clinical statement. In the meanwhile, the contrast media can also induce immune responses in the body. The signaling pathways involved in CI-AKI are closely associated with apoptosis, inflammation, and ROS production, and some studies suggest that these pathways may be potential targets for alleviating CI-AKI. The barrier is that, whatever bench or bed, it is hard to manage different kinds of cells from a certain kidney. Much more studies about cellular responses to contrast media need to be done. In conclusion, the pathogenesis of CI-AKI and the mechanism of DM as a predisposing factor for CI-AKI need further investigation in order to provide new clinical approaches to prevent and treat CI-AKI [19].

## References

[1] van der Molen A. J., Reimer P., Dekkers I. A. (2018). Post-contrast acute kidney injury—part 1: definition, clinical features, incidence, role of contrast medium and risk factors. *European Radiology*.

[2] Stacul F., van der Molen A. J., van der Molen A. J. (2011). Contrast induced nephropathy: updated ESUR contrast media safety committee guidelines. *European Radiology*.

[3] Andreucci M., Solomon R., Tasanarong A. (2014). Side effects of radiographic contrast media: pathogenesis, risk factors, and prevention. *BioMed Research International*.

[4] Garfinkle M. A., Stewart S., Basi R. (2015). Incidence of CT contrast agent-induced nephropathy: toward a more accurate estimation. *American Journal of Roentgenology*.

[5] Thomas M. E., Blaine C., Dawnay A. (2015). The definition of acute kidney injury and its use in practice. *Kidney International*.

[6] Fliser D., Fliser D., Laville M. (2012). A european renal best practice (ERBP) position statement on the kidney disease improving global outcomes (KDIGO) clinical practice guidelines on acute kidney injury: part 1: definitions, conservative management and contrast-induced nephropathy. *Nephrology, Dialysis, Transplantation: Official Publication of the European Dialysis and Transplant Association—European Renal Association*.

[7] Nash K., Hafeez A., Hou S. (2002). Hospital-acquired renal insufficiency. *American Journal of Kidney Diseases*.

[8] Gleeson T. G., Bulugahapitiya S. (2004). Contrast-induced nephropathy. *American Journal of Roentgenology*.

[9] Hose E. A., Kellum J. A., Selby N. M. (2018). Global epidemiology and outcomes of acute kidney injury. *Nature Reviews Nephrology*.

[10] van der Molen A. J., Reimer P., Dekkers I. A. (2018). Post-contrast acute kidney injury. Part 2: risk stratification, role of hydration and other prophylactic measures, patients taking metformin and chronic dialysis patients. *European Radiology*.

[11] Katzberg R. W., Newhouse J. H. (2010). Intravenous contrast medium-induced nephrotoxicity: is the medical risk really as great as we have come to believe?. *Radiology*.

[12] Mehran R., Nikolsky E. (2006). Contrast-induced nephropathy: definition, epidemiology, and patients at risk. *Kidney International. Supplement*.

[13] Heyman S. N., Rosen S., Khamaisi M., Miyata T., Eckard K.-U., Nangaku M. (2011). Hypoxia, oxidative stress, and the pathophysiology of contrast-media-induced nephropathy. *Oxidative Stress in Basic Research and Clinical Practice: Studies on Renal Disorders*.

[14] Heyman S. N., Rosen S., Khamaisi M., Idée J.-M., Rosenberger C. (2010). Reactive oxygen species and the pathogenesis of radiocontrast-induced nephropathy. *Investigative Radiology*.

[15] Zhang Y., Wang J., Yang X. (2012). The serial effect of iodinated contrast media on renal hemodynamics and oxygenation as evaluated by ASL and BOLD MRI effect of iodinated contrast media on renal hemodynamics and oxygenation as evaluated by ASL and BOLD MRI. *Contrast Media & Molecular Imaging*.

[16] Rosenberger C., Khamaisi M., Abassi Z. (2008). Adaptation to hypoxia in the diabetic rat kidney. *Kidney International*.

[17] Sies H., Berndt C., Jones D. P. (2017). Oxidative stress. *Annual Review of Biochemistry*.

[18] Caiazza A., Russo L., Sabbatini M., Russo D. (2014). Hemodynamic and tubular changes induced by contrast media. *BioMed Research International*.

[19] Sendeski M. M. (2011). Pathophysiology of renal tissue damage by iodinated contrast media. *Clinical and Experimental Pharmacology and Physiology*.

[20] Yang D., Yang D. (2013). Role of intracellular Ca^2+^ and Na^+^/Ca^2+^ exchanger in the pathogenesis of contrast-induced acute kidney injury. *BioMed Research International*.

[21] Anderson K. J., Christensen E. I., Vik H. (1994). Effects of iodinated X-ray contrast media on renal epithelial cells in culture. *Investigative Radiology*.

[22] Zager R. A., Johnson A. C. M., Hanson S. Y. (2003). Radiographic contrast media-induced tubular injury: evaluation of oxidant stress and plasma membrane integrity. *Kidney International*.

[23] Heyman S. N., Rosenberger C., Rosen S., Khamaisi M. (2013). Why is diabetes mellitus a risk factor for contrast-induced nephropathy?. *BioMed Research International*.

[24] Morabito S., Pistolesi V., Benedetti G. (2012). Incidence of contrast-induced acute kidney injury associated with diagnostic or interventional coronary angiography. *Journal of Nephrology*.

[25] Martin-Mateo M. C., Sanchez-Portugal M., Sanchez-Portugal M., Iglesias S., de Paula A., Bustamante J. (1999). Clinical study: oxidative stress in chronic renal failure. *Renal Failure*.

[26] Okamura D. M., Pennathur S., Pasichnyk K. (2009). CD36 regulates oxidative stress and inflammation in hypercholesterolemic CKD. *Journal of the American Society of Nephrology*.

[27] Evans R. G., Ince C., Joles J. A. (2013). Haemodynamic influences on kidney oxygenation: clinical implications of integrative physiology. *Clinical and Experimental Pharmacology and Physiology*.

[28] Wong P. C. Y., Li Z., Guo J., Zhang A. (2012). Pathophysiology of contrast-induced nephropathy. *International Journal of Cardiology*.

[29] Palm F., Nordquist L., Christopher S. W., Hansell P., Miyata T., Eckard K.-U., Nangaku M. (2011). Oxidative stress and hypoxia in the pathogenesis of diabetic nephropathy. *Oxidative Stress in Basic Research and Clinical Practice: Studies on Renal Disorders*.

[30] Lemineur T., Deby-Dupont G., Preiser J.-C. (2006). Biomarkers of oxidative stress in critically ill patients: what should be measured, when and how?. *Current Opinion in Clinical Nutrition and Metabolic Care*.

[31] Clark B. A., Kim D., Epstein F. H. (1997). Endothelin and atrial natriuretic peptide levels following radiocontrast exposure in humans. *American Journal of Kidney Diseases*.

[32] Khamaisi M., Raz I., Shilo V. (2008). Diabetes and radiocontrast media increase endothelin converting enzyme-1 in the kidney. *Kidney International*.

[33] Hansen P. B., Schnermann J. (2003). Vasoconstrictor and vasodilator effects of adenosine in the kidney. *American Journal of Physiology-Renal Physiology*.

[34] Nechemia-Arbeli Y., Khamaisi M., Rosenberger C. (2013). In vivo evidence suggesting reciprocal renal HIF-1 up-regulation and STAT3 activation in response to hypoxic and non-hypoxic stimuli. *Clinical and Experimental Pharmacology and Physiology*.

[35] Takenaka T., Inoue T., Ohno Y. (2012). Elucidating mechanisms underlying altered renal autoregulation in diabetes. *American Journal of Physiology-Regulatory, Integrative and Comparative Physiology*.

[36] Vallon V. (2011). The proximal tubule in the pathophysiology of the diabetic kidney. *American Journal of Physiology-Regulatory, Integrative and Comparative Physiology*.

[37] Juncos R., Garvin J. L. (2005). Superoxide enhances Na-K-2Cl cotransporter activity in the thick ascending limb. *American Journal of Physiology-Renal Physiology*.

[38] Epstein F. H., Silva P., Spokes K., Brezis M., Rosen S. (1989). Renal medullary Na-K-ATPase and hypoxic injury in perfused rat kidneys. *Kidney International*.

[39] Pickup J. C., Chusney G. D., Thomas S. M., Burt D. (2000). Plasma interleukin-6, tumour necrosis factor *α* and blood cytokine production in type 2 diabetes. *Life Sciences*.

[40] Bruno G., Merletti F., Biggeri A. (2003). Progression to overt nephropathy in type 2 diabetes: the casale monferrato study. *Diabetes Care*.

[41] Navarro J. F., Mora C., Macıéa M., Garcıéa J. (2003). Inflammatory parameters are independently associated with urinary albumin in type 2 diabetes mellitus. *American Journal of Kidney Diseases*.

[42] Hasegawa G., Nakano K., Sawada M. (1991). Possible role of tumor necrosis factor and interleukin-1 in the development of diabetic nephropathy. *Kidney International*.

[43] Dong X., Swaminathan S., Bachman L. A. (2007). Resident dendritic cells are the predominant TNF-secreting cell in early renal ischemia-reperfusion injury. *Kidney International*.

[44] Bertani T., Abbate M., Zoja C. (1989). Tumor necrosis factor induces glomerular damage in the rabbit. *The American Journal of Pathology*.

[45] Boyle J. J., Weissberg P. L., Bennett M. R. (2003). Tumor necrosis factor-*α* promotes macrophage-induced vascular smooth muscle cell apoptosis by direct and autocrine mechanisms. *Arteriosclerosis, Thrombosis, and Vascular Biology*.

[46] Koike N., Takamura T., Kaneko S. (2007). Induction of reactive oxygen species from isolated rat glomeruli by protein kinase C activation and TNF-*α* stimulation, and effects of a phosphodiesterase inhibitor. *Life Sciences*.

[47] De la Fuente M. (2002). Effects of antioxidants on immune system ageing. *European Journal of Clinical Nutrition*.

[48] Hardeman M. R., Goedhart P., Koen I. Y. (1991). The effect of low-osmolar ionic and nonionic contrast media on human blood viscosity, erythrocyte morphology, and aggregation behavior. *Investigative Radiology*.

[49] Lee D., Kim C. E., Park S. Y. (2018). Protective effect of artemisia argyi and its flavonoid constituents against contrast-induced cytotoxicity by iodixanol in LLC-PK1 cell. *International Journal of Molecular Sciences*.

[50] Miyauchi K., Takiyama Y., Honjyo J., Tateno M., Haneda M. (2009). Upregulated IL-18 expression in type 2 diabetic subjects with nephropathy: TGF-*β*1 enhanced IL-18 expression in human renal proximal tubular epithelial cells. *Diabetes Research and Clinical Practice*.

[51] Kilari S., Yang B., Sharma A., McCall D. L., Misra S. (2018). Increased transforming growth factor beta (TGF-*β*) and pSMAD3 signaling in a murine model for contrast induced kidney injury. *Scientific Reports*.

[52] Yaribeygi H., Atkin S. L., Sahebkar A. (2019). Interleukin-18 and diabetic nephropathy: a review. *Journal of Cellular Physiology*.

[53] Mahadevan P., Larkins R. G., Fraser J. R. E., Fosang A. J., Dunlop M. E. (1995). Increased hyaluronan production in the glomeruli from diabetic rats: a link between glucose-induced prostaglandin production and reduced sulphated proteoglycan. *Diabetologia*.

[54] Coleman D. L., Ruef C. (1992). Interleukin-6: an autocrine regulator of mesangial cell growth. *Kidney International*.

[55] Onk D., Onk A. O., Turkmen K. (2016). Melatonin attenuates contrast-induced nephropathy in diabetic rats: the role of interleukin-33 and oxidative stress. *Mediators of Inflammation*.

[56] Oweis A., Alshelleh S., Daoud A., Smadi M., Alzoubi K. (2018). Inflammatory milieu in contrast-induced nephropathy: a prospective single-center study. *International Journal of Nephrology and Renovascular Disease*.

[57] Wang S., Li Y., Fan J. (2017). Interleukin-22 ameliorated renal injury and fibrosis in diabetic nephropathy through inhibition of NLRP3 inflammasome activation. *Cell Death and Disease*.

[58] Kanaski K., Taduri G., Koya D. (2013). Diabetic nephropathy: the role of inflammation in fibroblast activation and kidney fibrosis. *Frontiers Endocrinology (Lausanne)*.

[59] Wada J., Makino H. (2016). Innate immunity in diabetes and diabetic nephropathy. *Nature Reviews Nephrology*.

[60] Pillon N. J., Azizi P. M., Li Y. E. (2015). Palmitate-induced inflammatory pathways in human adipose microvascular endothelial cells promotes monocyte adhesion and impairs insulin transcytosis. *American Journal of Physiology-Endocrinology and Metabolism*.

[61] Rajamani U., Jialal I. (2014). Hyperglycemia induces toll-like receptor-2 and -4 expression and activity in human microvascular retinal endothelial cells: implications for diabetic retinopathy. *Journal of Diabetes Research*.

[62] Li J., Jin C., Cleveland J. C. (2010). Enhanced inflammatory responses to toll-like receptor 2/4 stimulation in type 1 diabetic coronary artery endothelial cells: the effect of insulin. *Cardiovascular Diabetology*.

[63] Tang S. C. W., Leung J. C. K., Lai K. N. (2011). Diabetic tubulopathy: an emerging entity. *Contributions to Nephrology*.

[64] Akhter N., Madhoun A., Arefanian H. (2019). Oxidative stress induces expression of the toll-like receptors (TLRs) 2 and 4 in the human peripheral blood mononuclear cells: implications for metabolic inflammation. *Cellular Physiology and Biochemistry: International Journal of Experimental Cellular Physiology, Biochemistry, and Pharmacology*.

[65] Paraskevi P., Vassilios L., Theodoros E. (2017). Oxidative stress and acute kidney injury in critical illness pathophysiologic mechanisms—biomarkers—interventions, and future perspectives. *Oxidative Medicine and Cellular Longevity*.

[66] Dowling J. K., O’Neill L. A. J. (2012). Biochemical regulation of the inflammasome. *Critical Reviews in Biochemistry and Molecular Biology*.

[67] Gill R., Tsung A., Billiar T. (2010). Linking oxidative stress to inflammation: toll-like receptors. *Free Radical Biology and Medicine*.

[68] Rosin D. L., Okusa M. D. (2011). Dangers within: DAMP responses to damage and cell death in kidney disease. *Journal of the American Society of Nephrology*.

[69] Arend W. P., Palmer G., Gabay C. (2008). IL-1, IL-18, and IL-33 families of cytokines. *Immunological Reviews*.

[70] Lau A., Chung H., Komada T. (2018). Renal immune surveillance and dipeptidase-1 contribute to contrast-induced acute kidney injury. *Journal of Clinical Investigation*.

[71] Yaribeygi H., Atkin S. L., Simental-Mendía L. E., Barreto G. E., Sahebkar A. (2019). Anti-inflammatory effects of resolvins in diabetic nephropathy: mechanistic pathways. *Journal of Cellular Physiology*.

[72] Yano T., Itoh Y., Sendo T., Kubota T., Oishi R. (2003). Cyclic AMP reverses radiocontrast media-induced apoptosis in LLC-PK1 cells by activating a kinase/PI3 kinase. *Kidney International*.

[73] Saito M., Itoh Y., Yano T. (2003). Roles of intracellular Ca^2+^ and cyclic AMP in mast cell histamine release induced by radiographic contrast media. *Naunyn-Schmiedeberg’s Archives of Pharmacology*.

[74] Yano T., Itoh Y., Kubota T., Sendo T., Oishi R. (2004). A prostacyclin analog beraprost sodium attenuates radiocontrast media-induced LLC-PK1 cells injury. *Kidney International*.

[75] Andreucci M., Lucisano G., Faga T. (2011). Differential activation of signaling pathways involved in cell death, survival and inflammation by radiocontrast media in human renal proximal tubular cellsfferential activation of signaling pathways involved in cell death, survival and inflammation by radiocontrast media in human renal proximal tubular cells. *Toxicological Sciences*.

[76] Andreucci M., Faga T., Russo D. (2014). Differential activation of signaling pathways by low-osmolar and iso-osmolar radiocontrast agents in human renal tubular cells. *Journal of Cellular Biochemistry*.

[77] Gong X., Duan Y., Zheng J. (2016). Nephroprotective effects of N-acetylcysteine amide against contrast-induced nephropathy through upregulating thioredoxin-1, inhibiting ASK1/p38MAPK pathway, and suppressing oxidative stress and apoptosis in rats. *Oxidative Medicine and Cellular Longevity*.

[78] Wang Y., Zhang Z. Z., Wu Y., Ke J. J., He X. H., Wang Y. L. (2013). Quercetin postconditioning attenuates myocardial ischemia/reperfusion injury in rats through the PI3K/Akt pathway. *Brazilian Journal of Medical and Biological Research*.

[79] Tongqiang L., Shaopeng L., Xiaofang Y. (2016). Salvianolic acid B prevents iodinated contrast media-induced acute renal injury in rats via the PI3K/Akt/Nrf2 pathway. *Oxidative Medicine and Cellular Longevity*.

[80] Zhao Z., Liao G., Zhou Q. (2016). Sulforaphane attenuates contrast-induced nephropathy in rats via Nrf2/HO-1 pathway. *Oxidative Medicine and Cellular Longevity*.

[81] Chistiakov D. A., Orekhov A. N., Bobryshev Y. V. (2017). The impact of FOXO-1 to cardiac pathology in diabetes mellitus and diabetes-related metabolic abnormalities. *International Journal of Cardiology*.

[82] Shan L., Yang D., Zhu D., Feng F., Li X. (2019). High glucose promotes annulus fibrosus cell apoptosis through activating the JNK and p38 MAPK pathways. *Bioscience Reports*.

[83] Nishikawa T., Edelstein D., Du X. L. (2000). Normalizing mitochondrial superoxide production blocks three pathways of hyperglycaemic damage. *Nature*.

[84] Badal S. S., Danesh F. R. (2014). New insights into molecular mechanisms of diabetic kidney disease. *American Journal of Kidney Diseases*.

[85] Noh H., King G. L. (2007). The role of protein kinase C activation in diabetic nephropathy. *Kidney International*.

[86] Koya D., Jirousek M. R., Lin Y. W., Ishii H., Kuboki K., King G. L. (1997). Characterization of protein kinase C beta isoform activation on the gene expression of transforming growth factor-beta, extracellular matrix components, and prostanoids in the glomeruli of diabetic rats. *Journal of Clinical Investigation*.

[87] Bierhaus A., Schiekofer S., Schwaninger M. (2001). Diabetes-associated sustained activation of the transcription factor nuclear factor-*κ*B. *Diabetes*.

[88] Barnes P. J., Karin M. (1997). Nuclear factor-*κ*b—a pivotal transcription factor in chronic inflammatory diseases. *New England Journal of Medicine*.

[89] Moi P., Chan K., Asunis I., Cao A., Kan Y. W. (1994). Isolation of NF-E2-related factor 2 (Nrf2), a NF-E2-like basic leucine zipper transcriptional activator that binds to the tandem NF-E2/AP1 repeat of the beta-globin locus control region. *Proceedings of the National Academy of Sciences*.

[90] Khaleel S. A., Raslan N. A., Alzokaky A. A. (2019). Contrast media (meglumine diatrizoate) aggravates renal inflammation, oxidative DNA damage and apoptosis in diabetic rats which is restored by sulforaphane through Nrf2/HO-1 reactivation. *Chemico-Biological Interactions*.

